# Impact of COVID-19 on pediatric emergencies and hospitalizations in Singapore

**DOI:** 10.1186/s12887-020-02469-z

**Published:** 2020-12-23

**Authors:** Shu-Ling Chong, Jenifer Shui Lian Soo, John Carson Allen, Sashikumar Ganapathy, Khai Pin Lee, Arif Tyebally, Chee Fu Yung, Koh Cheng Thoon, Yong Hong Ng, Jean Yin Oh, Oon Hoe Teoh, Yee Hui Mok, Yoke Hwee Chan

**Affiliations:** 1 Department of Emergency Medicine, KK Women’s and Children’s Hospital, 100, Bukit Timah Road, Singapore, 229899 Singapore; 2 Duke-NUS Medical School, Singapore, Singapore; 3 Division of Medicine, KK Women’s and Children’s Hospital, Singapore, Singapore; 4 Centre for Quantitative Medicine, Duke-NUS Medical School, Singapore, Singapore; 5 Department of KK Women’s and Children’s Hospital, Infectious Disease Service, Singapore, Singapore; 6 Department of Paediatrics, KK Women’s and Children’s Hospital, Singapore, Singapore; 7 Children’s Intensive Care Unit, Department of Paediatric Subspecialties, KK Women’s and Children’s Hospital, Singapore, Singapore

**Keywords:** COVID-19, Child, Health services, Resource utilization

## Abstract

**Background:**

Coronavirus disease 2019 (COVID-19) has impacted the provision of health services in all specialties. We aim to study the impact of COVID-19 on the utilization of pediatric hospital services including emergency department (ED) attendances, hospitalizations, diagnostic categories and resource utilization in Singapore.

**Methods:**

We performed a retrospective review of ED attendances and hospital admissions among children < 18 years old from January 1st to August 8th 2020 in a major pediatric hospital in Singapore. Data were analyzed in the following time periods: Pre-lockdown (divided by the change in Disease Outbreak Response System Condition (DORSCON) level), during-lockdown and post-lockdown. We presented the data using proportions and percentage change in mean counts per day with the corresponding 95% confidence intervals (CIs).

**Results:**

We attended to 58,367 children with a mean age of 5.1 years (standard deviation, SD 4.6). The mean ED attendance decreased by 331 children/day during lockdown compared to baseline (*p* < 0.001), attributed largely to a drop in respiratory (% change − 87.9, 95% CI − 89.3 to − 86.3, *p* < 0.001) and gastrointestinal infections (% change − 72.4, 95%CI − 75.9 to − 68.4, *p* < 0.001). Trauma-related diagnoses decreased at a slower rate across the same periods (% change − 40.0, 95%CI − 44.3 to − 35.3, *p* < 0.001). We saw 226 children with child abuse, with a greater proportion of total attendance seen post-lockdown (79, 0.6%) compared to baseline (36, 0.2%) (p < 0.001). In terms of ED resource utilization, there was a decrease in the overall mean number of procedures performed per day during the lockdown compared to baseline, driven largely by a reduction in blood investigations (% change − 73.9, 95%CI − 75.9 to − 71.7, *p* < 0.001).

**Conclusions:**

We highlighted a significant decrease in infection-related presentations likely attributed to the lockdown and showed that the relative proportion of trauma-related attendances increased. By describing the impact of COVID-19 on health services, we report important trends that may provide guidance when planning resources for future pandemics.

**Supplementary Information:**

The online version contains supplementary material available at 10.1186/s12887-020-02469-z.

## Background

Coronavirus-19 disease (COVID-19), caused by SARS-CoV-2, was declared a pandemic by the World Health Organization (WHO) on March 11th, 2020 [1]. To date, it has claimed more than 1 million lives and infected more than 50 million worldwide [2]. COVID-19 has impacted the way medicine is practiced and the provision of health services in all specialties [3–6].

A strong effect of age on disease severity and mortality has consistently been shown since the early phases of the pandemic [7, 8]. A systematic review of 1065 children infected with SARS-CoV-2 demonstrated uniformly mild disease, presenting mostly with mild respiratory symptoms [9]. Nevertheless, the impact of COVID-19 on children and their families has been keenly felt throughout the world, not only because of the disease itself but also because of broad public health measures especially lockdowns being employed to control the pandemic. Lockdowns have consequent effect on social interactions amongst children and their families, on their education and access to various social and health services [10].

While the COVID-19 literature has provided a greater understanding on the course of the disease itself, important gaps in our knowledge have been highlighted [11]. Because of the far reaching effects on healthcare systems worldwide, it is equally important to study the effect of this pandemic on the epidemiological burden of diseases in general and their presentation to healthcare services, beyond patients with COVID-19 disease [11]. Beyond the impact on the landscape of acute diseases, the impact of COVID-19 on mental health has also been reported among adults [12, 13]. The psychological impact of COVID-19 on the pediatric population and family health has not been well reported to date and deserves closer study [14, 15]. Specifically, the effect of the pandemic on rates of domestic violence and child abuse requires investigation.

Singapore employs a 4-tier color coded Disease Outbreak Response System Condition (DORSCON) system in escalating order of green, yellow, orange and red, to categorize the severity of a pandemic and to signal policy changes to the community (Table 1) [16]. Following the first case of COVID-19 in Singapore on January 23rd 2020 [17] and evidence of locally-transmitted clusters, the Ministry of Health raised the pandemic response level to Orange on February 7th 2020 [18]. This translated to widespread temperature, restriction of movement between schools and institutions, and segregating the care of patients with respiratory infections from other patients in healthcare institutions [19]. The World Health Organization (WHO) subsequently declared COVID-19 a pandemic on March 11th 2020 [20]. When the number of infections rose to about a thousand community cases [21], Singapore underwent a nation-wide lockdown, termed the “community circuit breaker” [22] between April 7th and June 1st 2020. Only essential services like healthcare, social services, financial services, water and energy related services were permitted to operate. All educational institutions and child care services were closed. The public was strictly discouraged from leaving the home except to obtain essential household supplies and to seek medical attention. Post lockdown, the public is currently strongly advised to maintain safe social distancing, and wear masks at all times when outside the home [23].

**Table 1:**
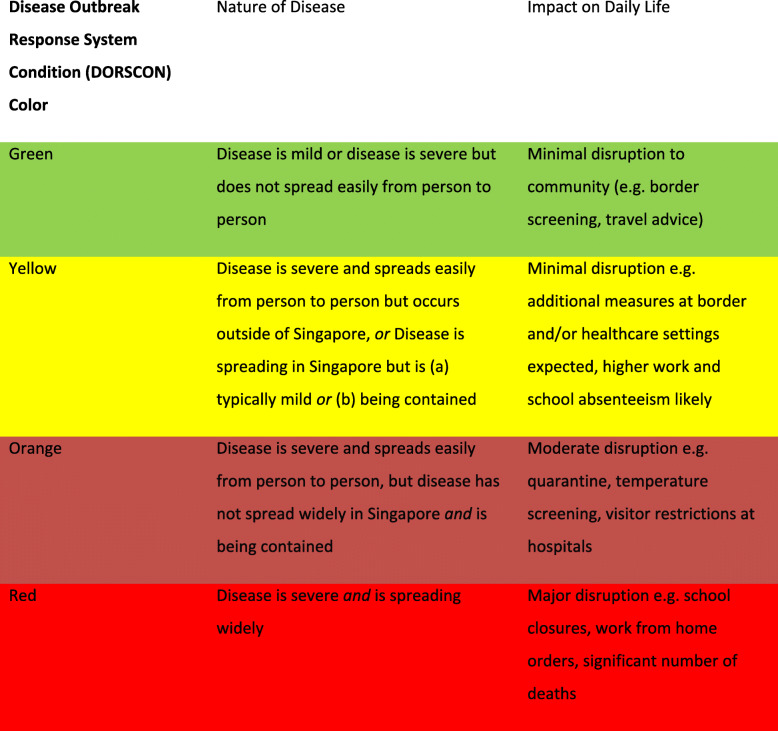
Disease Outbreak Response System Condition (DORSCON) categories by color

**Reference** [16]:

We aimed to study the impact of COVID-19 on the utilization of acute pediatric hospital services including emergency care, hospitalizations, diagnostic categories and resource utilization. Analysis was performed in relation to 3 key time points: raising of the pandemic response level to DORSCON orange, implementation of lockdown and lifting of major lockdown measures in Singapore.

## Methods

We performed a retrospective review of both ED and hospitalization electronic health records among children < 18 years old. KK Women’s and Children’s Hospital is an 830-bed institution with 500 pediatric and neonatal beds in Singapore that caters to mothers and children, with an annual ED attendance of about 150,000 children. It was primarily designated by the Ministry of Health to screen and treat children with COVID-19. We divided the study period from January 1st to August 8th 2020, by the following time periods: Pre-lockdown was divided into pre- and post-DORSCON orange: Pre-DORSCON orange was defined from January 1st to February 6th 2020 and post-DORSCON orange from February 7th to April 6th [19]. During-lockdown was defined as April 7th to June 1st 2020 [22] and post-lockdown period was June 2nd to August 8th 2020 [23].

We obtained patient demographics, ED triage status and management. In our ED, we triage patients based on their vital signs and clinical appearance, with priority 1 (most critically ill), 2 and 3 (least critically ill), as prior validated [24]. Components of the local triage system are similar to the more widely used Emergency Severity Index (ESI) internationally, maintained by the Agency for Healthcare Research and Quality [25]. In our ED, all trauma cases are triaged at least as priority 2, to expedite subsequent procedural intervention. ED procedures were extracted based on electronically specified codes and categorized into resuscitation-related, non-trauma and trauma/surgical-related. Resuscitation-related procedures included cardiopulmonary resuscitation, tracheal intubation and fluid resuscitation. Non-trauma procedures were categorized as blood-, urine- and cannulation-related interventions. Trauma procedures were categorized into toilet and suture, manipulation and reduction, and procedural sedation. Procedural sedation is routinely carried out in our ED for common trauma procedures. The most common form of procedural sedation practiced in our ED is ketamine sedation [26]. We documented the ED disposition, and divided hospitalized patients into those admitted to the general ward (stable), high dependency (moderately ill) or intensive care unit (ICU) (most severely ill). We also recorded the overall hospital length of stay (LOS).

To study the clinical diagnoses, we used the SNOMED-Clinical Terms (SNOMED-CT) [27] for ED diagnoses and International Statistical Classification of Diseases and Related Health Problems (ICD 10-AM) diagnostic codes for inpatient diagnoses. Both are currently in use in our institution. The list of diagnoses specifically studied are found in the Supplementary Table.

### Statistical analysis

We compared the patient attendance, characteristics, management and disposition variables among the 4 time-period groups defined above. Categorical variables were presented using proportions and percentages and continuous variables as mean (standard deviation, SD) or median (interquartile range, IQR) depending on normality. The natural logarithm was used as a normalizing transformation on length of stay outcomes which exhibited right-skewed distributions. Categorical variables were analyzed using an overall chi-squared test involving the three groups followed by pair-wise comparisons. One-way ANOVA accommodating unequal variances among the three study groups was used to analyze continuous variables followed by post-hoc least significant difference (LSD) pair-wise comparisons. The Wilcoxon rank sum was performed for pair-wise comparisons of non-parametric data. A generalized linear model using a negative binomial count distribution with log link function was employed to analyze rate ratios of mean counts per day. We used rate ratios and the % change in rates, with their corresponding 95% confidence intervals (95%CI) to demonstrate the effect sizes between successive periods. The data analysis was performed using SAS software, version 9.4 of the SAS System (SAS Institute, Inc. Cary, NC, USA).

We obtained ethics approval from the SingHealth Centralised Institutional Review Board (2020/2760) with waiver of documented informed consent.

## Results

A total of 58,367 children were seen in the ED during the study period, had a mean age of 5.1 years (SD 4.6) and were mostly males (32,292, or 55.3%). The mean daily ED attendance was 264 patients (SD 39), with a median hospital stay of 2.0 days (IQR 1.0–3.0).

**Fig. 1:**
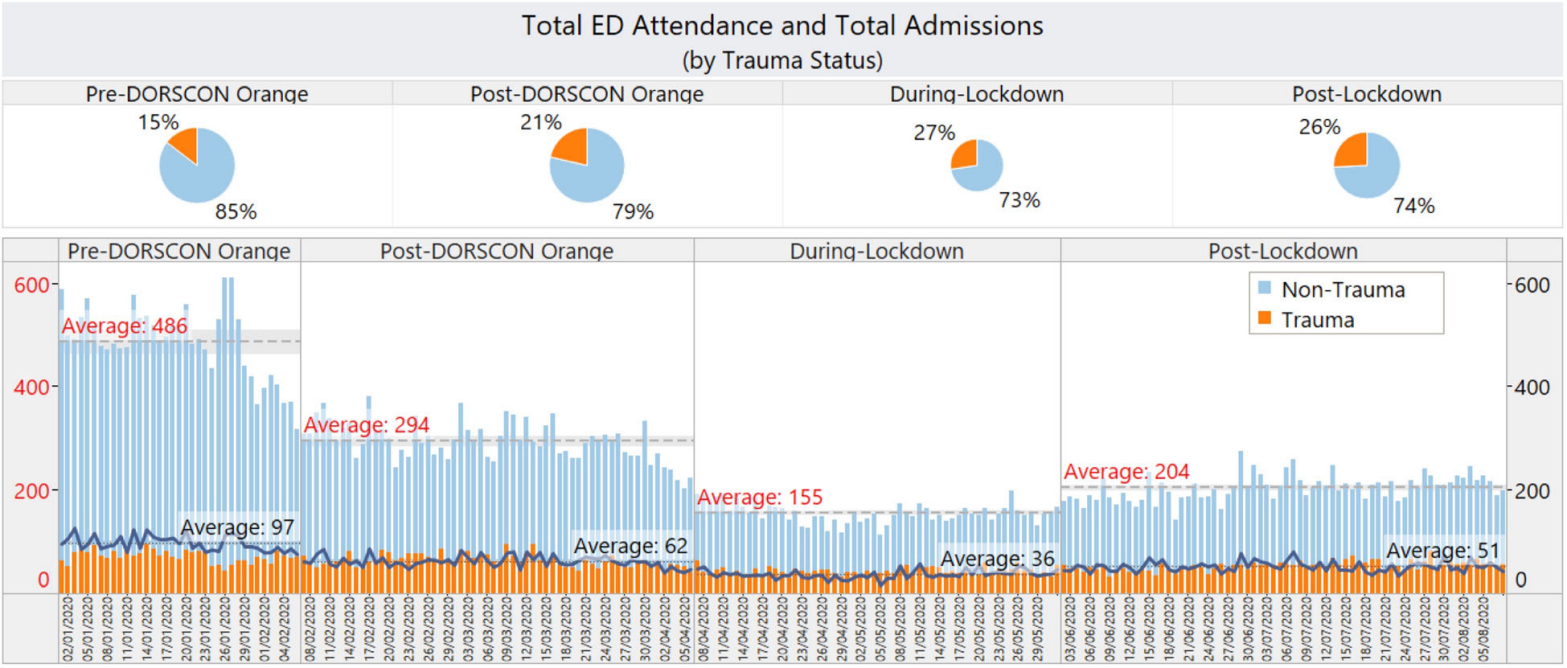
Trend of total ED attendance and total number of patients hospitalized. Bold line shows the daily number of hospitalizations

There was a large decrease in mean ED attendance of 331 children/day during lockdown compared to pre-DORSCON orange (*p* < 0.001). (Table 2 and Fig. 1) Children seen during the lockdown period (4.6 years, SD 4.4) were younger than pre-lockdown (5.0 years, SD 4.5) (p < 0.001). The proportion of Priority 1 patients remained between 2.0–2.8% throughout the study duration, and there were no significant differences in the proportion that were admitted to High Dependency (moderately ill) or ICU (severely ill). (Table 2). A total of 18 emergency codes were called. There were no differences in resuscitation procedures performed between the 4 time periods.

**Table 2:** Characteristics of patients presenting in each period

Variable	Pre-DORSCON Orange (1) *N* = 18,016	Post-DORSCON Orange (2) *N* = 17,698	During Lockdown (3) *N* = 8743	Post Lockdown (4) *N* = 13,910	p-value (pairwise comparisons)		
					(2) vs (1)	(3) vs (2)	(4) vs (3)
Mean number seen in the ED, per day (SD)	487 (70)	295 (38)	156 (20)	205 (25)	< 0.001	< 0.001	< 0.001
Age in years, mean (SD)	4.7 (4.3)	5.3 (4.6)	4.6 (4.4)	5.6 (4.8)	< 0.001	< 0.001	< 0001
Males, n (%)	10,021 (55.6)	9890 (55.9)	4772 (54.6)	7609 (54.7)	0.622	0.045	0.859
Triage status, n (%)							
Priority 1	498 (2.8)	429 (2.4)	171 (2.0)	335 (2.4)	< 0.001	< 0.001	0.024
Priority 2	9566 (53.1)	8782 (49.6)	4832 (55.3)	7507 (54.0)			
Priority 3	7952 (44.1)	8487 (48.0)	3740 (42.8)	6068 (43.6)			
Requiring procedures, n (%)	9224 (51.2)	10,202 (57.6)	5334 (61.0)	8423 (60.6)	< 0.001	< 0.001	0.495
Inpatient, n (%)							
General ward	3395 (96.9)	3484 (97.0)	1923 (96.0)	3315 (96.4)	0.608	0.141	0.611
High Dependency	85 (2.4)	89 (2.5)	67 (3.3)	108 (3.1)			
ICU	25 (0.7)	19 (0.5)	13 (0.7)	16 (0.5)			
Hospital LOS in days, mean (SD)	2.5 (3.6)	2.6 (4.8)	2.7 (4.0)	2.7 (3.3)	0.221†	0.012†	0.185†
Hospital LOS in days, median (IQR)	2.0 (1.0–3.0)	2.0 (1.0–3.0)	2.0 (1.0–3.0)	2.0 (1.0–3.0)	0.003	0.110	0.125

*LOS* Length of stay

† *P*-values from log-transformed analysis of LOS

**Fig. 2:**
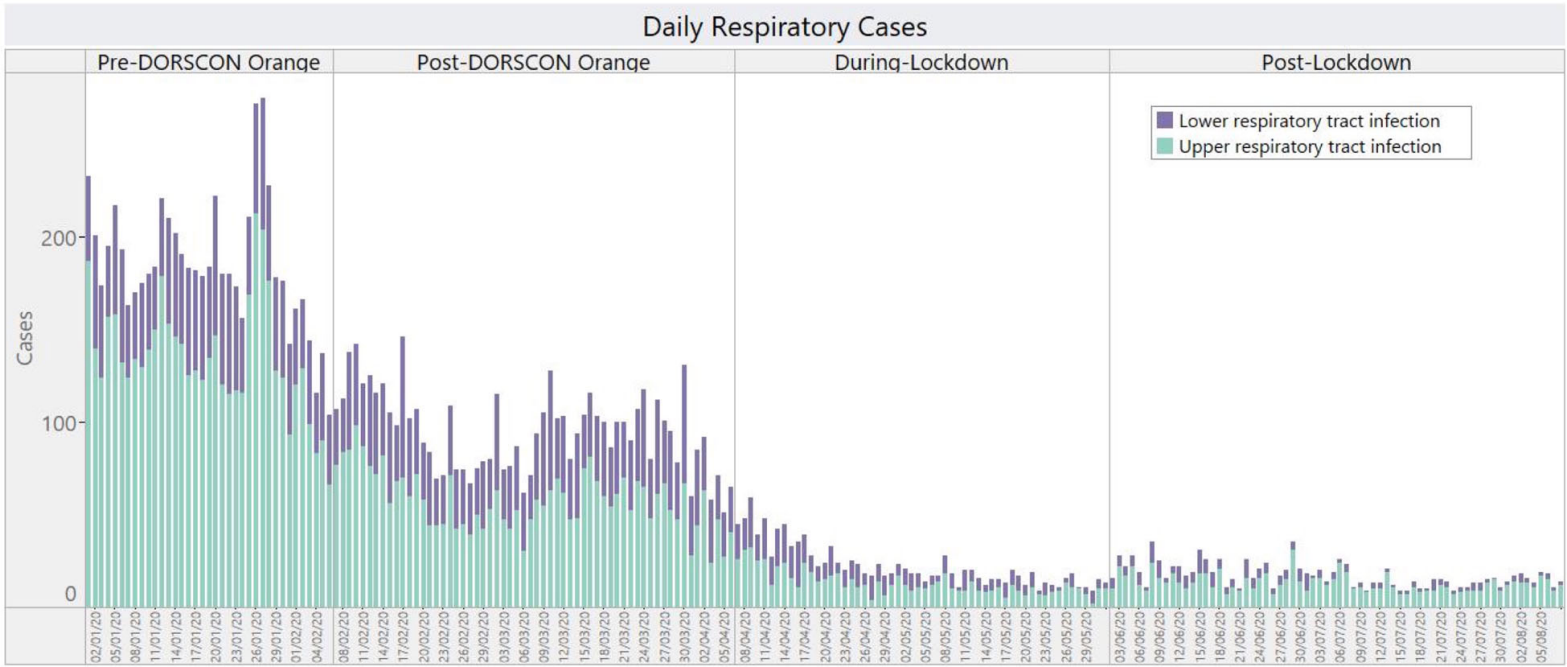
Trend of total, upper and lower respiratory tract infections

We saw significant reductions in common childhood infections with the progression of the pandemic (Table 3 and Fig. 2). During lockdown compared to pre-DORSCON orange, both respiratory infection rates (% change in mean number per day − 87.9, 95% CI − 89.3 to − 86.3, *p* < 0.001) and gastrointestinal infection rates (% change − 72.4, 95%CI − 75.9 to − 68.4, p < 0.001) dropped significantly. Hand foot mouth disease rates decreased more than five-fold in the same periods (% change − 95.4, 95% CI − 97.4 to − 92.0, *p* < 0.001). When the lockdown was lifted, lower respiratory tract infection rates continued to decrease (% change − 55.7, 95%CI − 64.6 to − 44.7, *p* < 0.001) while the decrease slowed down for upper respiratory tract infections (% change 0, 95% CI − 13.5 to + 15.7, *p* = 0.994) and gastrointestinal infections started to rise (% change + 44.7%, 95% CI 27.3 to 64.4, *p* < 0.001). (Table 3) Trauma-related diagnoses decreased at a slower rate during lockdown compared to pre-DORSCON orange (% change − 40.0, 95%CI − 44.3 to − 35.3, *p* < 0.001). More injuries occurred at home during lockdown (1601, 78.8%) compared to pre-lockdown (2685, 44.3%).

**Table 3:** Mean number of patients seen in the ED by diagnostic codes, per day

Mean number seen in the ED, per day (SD)	Pre-DORSCON Orange (1)	Post-DORSCON Orange (2)	During Lockdown (3)	Post Lockdown (4)	% change in mean number per day (95% CI)		
					(2) vs (1)	(3) vs (2)	(4) vs (3)
Total number of patients	487 (70)	295 (38)	156 (20)	205 (25)	−39.4 (−42.7, − 36.0)	−47.1 (− 49.5, − 44.5)	+ 31.0 (+ 25.5, + 36.8)
**Infection-related Diagnosis**							
Respiratory Tract Infections (all)	186 (36)	95 (22)	22 (11)	17 (6)	−48.7 (−53.0, − 44.0)	−76.5 (− 79.2, − 73.4)	−23.0 (− 33.4, − 11.0)
Upper respiratory	136 (31)	58 (16)	13 (7)	13 (5)	−57.3 (− 61.5, − 52.6)	−77.3 (− 80.1, − 74.1)	0 (−13.5, + 15.7)
Lower respiratory	50 (10)	37 (11)	9 (6)	4 (3)	−25.3 (− 32.3, − 17.5)	−75.1 (− 78.6, − 71.1)	−55.7 (− 64.6, − 44.7)
Gastroenteritis	27 (8)	12 (4)	8 (3)	11 (4)	−56.1 (− 61.2, − 50.3)	−37.1 (− 44.9, − 28.3)	+ 44.7 (+ 27.3, + 64.4)
Hand Foot Mouth Disease	5 (3)	2 (2)	0.3 (0.6)	0.4 (0.6)	−66.4 (− 74.3, − 56.2)	−86.4 (− 92.4, − 75.5)	+ 41.2 (− 30.1, + 185.0)
**Trauma-related diagnosis**	71 (12)	63 (13)	42 (7)	53 (9)	−11.1 (− 17.7, − 4.0)	− 32.5 (− 37.0, − 27.7)	+ 23.9 (+ 16.6, + 31.6)

*SD* standard deviation; 95%CI = 95% confidence interval

There was a decrease in the overall mean number of procedures performed per day during the lockdown compared to pre-DORSCON orange (% change − 61.8, 95%CI − 63.9 to − 59.6, *p* < 0.001), largely driven by a reduction in blood investigations (% change − 73.9, 95%CI − 75.9 to − 71.7, p < 0.001) (Table 4). Trauma-related procedures decreased at a slower rate and comprised a greater proportion of procedures during the lockdown. Children with lacerations requiring toilet and suture made up 6.5% of the procedures during the lockdown compared to 2.5% pre-DORSCON orange (*p* < 0.001).

**Table 4:** Mean number of procedures or sedations performed in the ED, per day

Mean number performed in the ED, per day (SD)	Pre-DORSCON Orange (1)	Post-DORSCON Orange (2)	During Lockdown (3)	Post Lockdown (4)	% change in mean number per day (95% CI)		
					[2] vs (1)	(3) vs (2)	(4) vs (3)
Any procedure	249 (34)	170 (23)	95 (14)	124 (17)	−31.8 (− 35.5, − 27.9)	−44.0 (− 46.8, − 41.0)	+ 30.0 (+ 23.7, + 36.7)
**Non-trauma related**							
Blood investigations	113 (24)	52 (10)	30 (6)	43 (8)	−54.2 (− 57.8, − 50.3)	−43.0 (− 47.1, − 38.6)	+ 43.8 (+ 34.3, + 53.8)
Urine investigations	49 (10)	31 (6)	24 (7)	31 (6)	−36.0 (− 41.2, − 30.5)	−22.6 (− 29.0, − 15.5)	+ 29.7 (+ 19.3, + 41.0)
Intravenous cannulation	15 (6)	6 (3)	5 (2)	8 (3)	−58.0 (− 64.3, − 50.6)	−20.4 (− 31.6, − 7.3)	+ 54.5 (+ 32.8, + 79.6)
**Trauma related**							
Toilet and suture	11 (3)	11 (4)	10 (3)	9 (3)	−0.3 (−11.9, + 12.8)	− 12.0 (− 22.0, − 0.8)	−7.6 (− 17.5, + 3.5)
Manipulation and reduction	3 (2)	4 (2)	2 (2)	3 (2)	+ 7.0 (−14.4, + 33.9)	−39.8 (− 52.0, − 24.6)	+ 32.6 (+ 4.6, + 68.1)
Procedural sedation	3 (2)	3 (2)	2 (1)	2 (2)	−10.5 (−30.1, + 14.7)	−17.9 (− 34.9, + 3.6)	+ 5.0 (− 18.1, + 34.3)

*95%CI* 95% confidence interval

We saw a total of 226 children with child abuse-related diagnoses during the study period. While the mean number remained constant at about 1 per day throughout the pandemic, these children constituted a greater proportion of children seen during lockdown (44, 0.5%) and post-lockdown (79, 0.6%) compared to pre-DORSCON orange (36, 0.2%) (p < 0.001). There were more males involved during lockdown (36, 81.8%) compared to pre-lockdown (52, 50.5%) and post-lockdown (44, 55.7%) (*p* = 0.005). Hospital stay, although longer during the lockdown (median LOS 7.0 days, IQR 2.0–13.0) compared to pre-DORSCON orange (median LOS 5.5 days, IQR 3.0–8.0), was not statistically significant (*p* = 0.855) (Table 5).

**Table 5:** Characteristics of patients diagnosed with child abuse related diagnoses in each period

Variable	Pre-DORSCON Orange (1) *N* = 18,016	Post-DORSCON Orange (2) *N* = 17,698	During Lockdown (3) *N* = 8743	Post Lockdown (4) *N* = 13,910	p-value (pairwise comparisons)		
					(2) vs (1)	(3) vs (2)	(4) vs (3)
Total number (%)	36 (0.2)	67 (0.4)	44 (0.5)	79 (0.6)	0.002	0.140	0.519
Age of child in years, mean (SD)	8.0 (4.7)	7.0 (4.3)	6.9 (4.2)	7.7 (4.4)	0.309	0.884	0.323
Males (%)	17 (47.2)	35 (52.2)	36 (81.8)	44 (55.7)	0.627	0.002	0.004
Hospital LOS in days, mean (SD)	6.6 (5.8)	5.6 (5.3)	8.4 (6.6)	6.1 (5.5)	0.376†	0.141†	0.485†
Hospital LOS in days, median (IQR)	5.5 (3.0–8.0)	3.0 (1.0–9.0)	7.0 (2.0–13.0)	4.0 (3.0–7.0)	0.892	0.492	0.900

*LOS* Length of stay

† *p*-values from log-transformed analysis of LOS

## Discussion

We report the effect of COVID-19 on the number and type of case presentations in a large tertiary pediatric institution in Singapore. We found a large decrease in ED attendance of more than 300 children a day, attributed largely to the decrease in infection-related attendances, in particular respiratory-related infections. There was a corresponding increase in the proportion of trauma-related cases.

The sharp decline in the overall number of cases seen during the pandemic is consistent with other published reports, which have attributed this phenomena to social distancing measures that reduced the rate of transmission of infections, as well as public fear of visiting healthcare institutions [28–30]. In our institution, this decline in attendance has not been seen in previous years and cannot be attributed to seasonal changes in ED attendances. We did not see a delayed increase in the proportion of Priority 1 (most ill) children late into the lockdown or early post-lockdown, that would suggest delayed health-seeking among ill children most deserving of medical attention. We hypothesized that the proportion of children with less severe disease would decrease, since we rationalized that most caregivers would shun the hospital given the pandemic. However, the proportion of children triaged as priority 3 (least ill) only decreased by a small proportion from pre-pandemic to lockdown (1.3%). We recognize that apart from the actual disease states, daily ED attendance is a dynamic play of current policy and health-seeking behavior. With each phase of the pandemic, the Ministry of Health (Singapore) continued to upscale Sars-CoV2 testing and recommended a low threshold to refer school-going children to tertiary hospitals for surveillance swabs [31]. These included children with mild respiratory symptoms, and would have contributed to the number of stable-appearing Priority 3 children.

We found a steep drop in infection-related attendances, particularly upper and lower respiratory infections, which was consistent with local national epidemiological data [32], as well as reported trends from other EDs [29]. Lockdown measures would have reduced the transmission of air-borne and droplet-transmitted diseases of all causes. Post-lockdown, social distancing and mandatory mask-wearing are likely to slow down the subsequent increase in respiratory infections [33, 34]. With the lifting of the lockdown and congregation of children in schools and recreation, rates of non-respiratory infections like gastroenteritis started to rise again.

We also expected the number of trauma-related procedures to decrease, since the lockdown would ensure that children stay at home, thus eliminating injuries that occur in school, on the road and in common areas e.g. recreation areas and playgrounds. However, the rate of decrease of injury-related complaints was slower than that of infection-related presentations, resulting in an increased proportion of children seen during lockdown who required procedures like manipulation for fractures and dislocations, or toilet and suture for lacerations. This reinforces the fact that the home is still the most common location for injuries [35], with the implication that we should consider upstream interventions involving home injury prevention education initiatives in future lockdown states. They also signal a need to allocate sufficient ED expertise, manpower and space to handle these injuries.

We found an increase in the proportion of child-abuse related complaints and diagnoses. We recognize that there is reduced visibility of vulnerable children in the community during a lockdown, hence rates of child abuse may have been under-reported. While psychiatric-related disorders attributed to the lockdown state have been widely reported in the adult and adolescent literature [36, 37], rates of domestic violence and child abuse in particular, have not been adequately studied [15]. Studies on child maltreatment during the pandemic have reported that parental factors including job loss, burnout, anxiety and depression could be contributory factors [38, 39]. This again, has practical implications on future lockdown states, where pediatric services must focus not only on managing the acutely unwell child, but also ensure that the mental and social wellbeing of families and children are cared for, particularly in vulnerable families [40]. Gaps that already exist between health care providers and the community become more apparent during a pandemic [41]. There is an urgent need to collaborate with social services, counsellors and other community-based resources to prevent such events from occurring.

### Limitations

We recognize that a retrospective study design would inadvertently include inaccuracies in diagnostic codes and documented resources. Although we had more than 2 months of post-lockdown data, we recognize that this may be insufficient to study trends that emerged post-lockdown. We also recognize that ED attendances would have been largely impacted by national policies that evolved through the pandemic and were difficult to account for in detail here. Nevertheless, we highlighted the time-points where major policy changes were made. Regarding the child abuse-related cases, we were not able to provide further detail on specific triggers that could have led to actionable recommendations. Finally, being a single-center study, we acknowledge that our findings require corroboration from other centers before they can be generalized.

## Conclusion

We described the impact of COVID-19 on health services in a major pediatric hospital in Singapore. We highlighted a significant decrease in infection-related presentations likely attributed to the lockdown and showed that the relative proportion of trauma-related attendances increased. Given the consistent number of children seen for child abuse-related complaints, there is an urgent need to consider the emotional wellbeing of families during lockdown states. These trends may provide guidance when planning resources for pandemics and future lockdown states.

## Supplementary Information

**Additional file 1.** Supplementary Table. Details of diagnostic codes. This is the list of diagnostic codes, by SNOMED-Clinical Terms (SNOMED-CT) and International Statistical Classification of Diseases and Related Health Problems (ICD 9 and 10-AM).

## Data Availability

The datasets used and/or analysed during the current study are available from the corresponding author on reasonable request.

## Abbreviations

COVID-19: Coronavirus disease 2019
DORSCON: Disease Outbreak Response System Condition
ED: Emergency Department
ICD: International Statistical Classification of Diseases and Related Health Problems
LOS: Length of stay
SNOMED-CT: SNOMED-Clinical Terms
